# ﻿Taxonomic notes on *Neocinnamomum* (Lauraceae): a new combination and a new species from southwestern China

**DOI:** 10.3897/phytokeys.269.177163

**Published:** 2026-01-16

**Authors:** Wei-Lan Lin, Zi-Yi Zhang, Chao-Yi Deng, Wen-Bin Xu, Bing Liu

**Affiliations:** 1 State Key Laboratory of Plant Diversity and Specialty Crops, Institute of Botany, Chinese Academy of Sciences, Beijing 100093, China Institute of Botany, Chinese Academy of Sciences Beijing China; 2 University of Chinese Academy of Sciences, Beijing 101408, China University of Chinese Academy of Sciences Beijing China; 3 Agriculture and Forestry Institute of Southwestern Guizhou, Xingyi 562400, China Agriculture and Forestry Institute of Southwestern Guizhou Xingyi China; 4 Wuhan Botanical Garden, Chinese Academy of Sciences, Wuhan 430074, China Wuhan Botanical Garden, Chinese Academy of Sciences Wuhan China; 5 Sino-Africa Joint Research Center, Chinese Academy of Sciences, Wuhan 430074, China Sino-Africa Joint Research Center, Chinese Academy of Sciences Wuhan China

**Keywords:** China, *
Neocinnamomum
*, new combination, new species, taxonomy

## Abstract

Here, *Neocinnamomum macrocarpum* (Wen B. Xu & B.S. Xia) Bing Liu, Wei L. Lin & Wen B. Xu and *Neocinnamomum citratum* C.Y. Deng, Bing Liu & Wei L. Lin from southwestern China are described as a new combination and a new species, respectively. The new species, *N. citratum*, differs from all other species of *Neocinnamomum* by the strong lemon fragrance of its branches, leaves, and bark. It is closely related to *N. lecomtei* but is distinguished by the sparsely white sericeous leaf blade on the abaxial surface when young (vs. densely rusty pubescent on both surfaces when young and abaxially when mature) and by oblate to globose fruits (vs. ellipsoid fruits in the latter species). The new combination, *N. macrocarpum*, is similar to *N. caudatum* in having puberulent branchlets, leaves glabrous on both surfaces, transverse veins subhorizontal and subparallel in arrangement, and fruits that are red or dark purple when ripe. However, it differs from the latter species by having triplinerved leaves, axillary cymules, a panicle that is terminal or occasionally axillary but underdeveloped, small flowers 3–4 mm in diameter, and widely ovoid fruits (vs. leaves trinerved, panicles terminal and axillary with a well-developed rachis, flowers 6–8 mm in diameter, and narrowly ellipsoid fruits in the latter species).

## ﻿Introduction

The family Lauraceae is the largest family in the clade magnoliids, currently containing 63 genera and more than 3,300 species, of which only the genus *Cassytha* L. is a semiparasitic twiner, while the rest are trees or shrubs (Rohwer 1993; Li et al. 2025). Lauraceae are widely distributed in tropical and subtropical regions of the world, with the main centers of diversity located in tropical Asia and tropical America (Rohwer 1993; Chanderbali et al. 2001). There are about 25 genera and 445 species in China, including 316 endemic species, with a high endemism of 71%, many of which are narrowly distributed species (Li et al. 2008a).

*Neocinnamomum* H. Liu was established by Liu in 1934 based on the presence of four anther sacs arranged horizontally (Liu 1934). It is a small genus in the Perseeae group of the Lauraceae family, containing seven known species (including one variety) distributed in tropical and subtropical Asia (Kostermans 1974; Li et al. 1982, 2008b; Xu et al. 2017). Species of the genus are characterized as evergreen trees or shrubs; leaves alternate, with blades trinerved (the two lateral nerves arising from near the leaf blade base) or triplinerved (the two lateral nerves arising from the midrib above the leaf blade base by more than 0.5 cm); small cymules composed of one to multiple flowers, remotely arranged in an axillary or terminal panicle or solitary in leaf axils; flowers 3-merous, bisexual, with a very short perianth tube; anthers 4-celled, arranged in an almost transverse series; and enlarged cupules with persistent tepals, which together with the fruit form a gourd-like structure. The systematic position of *Neocinnamomum* within the Lauraceae family was not confirmed until recently. Previous studies have shown that *Neocinnamomum*, *Cassytha*, and *Caryodaphnopsis* are closely related and that all three represent isolated lineages within Lauraceae (Li et al. 2016; Song et al. 2017, 2020; Liu et al. 2021; Yang et al. 2023, 2025). Their systematic position lies between the tribe Cryptocaryeae and the core group of the Lauraceae family. Song et al. (2020) proposed a tribal classification for Lauraceae and established a new tribe, Neocinnamomeae, which consists of the single genus *Neocinnamomum* and was subsequently recognized by Li et al. (2025).

Several new members of *Neocinnamomum* were published after its establishment (Merrill 1934; Allen 1939; Kostermans 1974; Wei 1988; De Kok 2023). *Neocinnamomum caudatum* (Nees) Merr. was published by Merrill in 1934 based on *Cinnamomum caudatum* Nees and is characterized by numerous transverse veins on the leaf blade that are subhorizontal and subparallel, connected by remote vertical veinlets and forming a transversely elongate reticulum; trinerved leaves; inflorescences consisting of numerous, remote cymules arranged into a developed axillary or terminal panicle; and narrowly ellipsoid fruits, 1.5–2 × 0.8–1 cm, red when ripe (Merrill 1934; Li et al. 1982). In 2017, a variety, Neocinnamomum caudatum var. macrocarpum Wen B. Xu & B.S. Xia, was published based on specimens collected from Debao County, Guangxi Province, southwestern China (Xu et al. 2017). This variety differs from *N. caudatum* mainly by its triplinerved leaf blades and fruit shape.

We conducted field investigations in southwestern China (Guangxi, Yunnan, and Guizhou provinces) and collected a number of specimens bearing both flowers and fruits of *Neocinnamomum*. Our new investigations and observations have demonstrated the necessity of taxonomic changes. First, we observed significant differences between N. caudatum var. macrocarpum and N. caudatum var. caudatum, beyond fruit shape and leaf blade venation. This is corroborated by a recent plastome phylogenomic study, which showed that this variety alone forms a clade and is not nested within N. caudatum var. caudatum (Cao et al. 2023). Moreover, in addition to the type locality, we discovered multiple distribution locations of N. caudatum var. macrocarpum in Guangxi Province. We therefore elevate N. caudatum var. macrocarpum to species rank and name it *Neocinnamomum macrocarpum* (Wen B. Xu & B.S. Xia) Bing Liu, Wei L. Lin & Wen B. Xu. Second, we collected a notable species of *Neocinnamomum* that has a strong lemon fragrance in its branches and leaves, which clearly distinguishes it from other known species of the genus. Further morphological studies indicate that this taxon represents a new species of *Neocinnamomum*. As a result, we describe this species here as new to science.

## ﻿Materials and methods

### ﻿Morphology and anatomy

We conducted several field investigations in southern China, including Guangxi, Guizhou, Yunnan, and Hainan, in December 2024 and January and May 2025. Voucher specimens were collected, flowers were preserved in FAA for morphological observations, and leaf materials were dried with silica gel for DNA extraction. Photographs of vegetative and reproductive characters were taken using an Olympus EM One Mark II. Preserved flowers were dissected and observed, and photographs were taken under a stereo microscope (Leica S8 APO) at the State Key Laboratory of Plant Diversity and Specialty Crops, Institute of Botany, Chinese Academy of Sciences.

### ﻿Figure treatments

Line drawings were produced manually using black ink. Line drawings and figures showing morphological characters were edited and merged using Adobe Photoshop CS2 ver. 9.0. The distribution map was generated using ArcMap ver. 10.0 and Microsoft Paint (Windows 10).

## ﻿Taxonomic treatment

### ﻿ Neocinnamomum macrocarpum

Taxon classificationPlantaeLauralesLauraceae

(Wen B.Xu & B.S.Xia) Bing Liu, Wei L. Lin & Wen B. Xu, comb. et stat. nov.

A8F987F7-6AC1-56C5-B124-55E8CE02F27F

urn:lsid:ipni.org:names:77375095-1

Fig. 1

#### Basionym.

Neocinnamomum caudatum var. macrocarpum Wen B. Xu & B.S. Xia, Guihaia, 37: 856. 2017.

**Figure 1.**
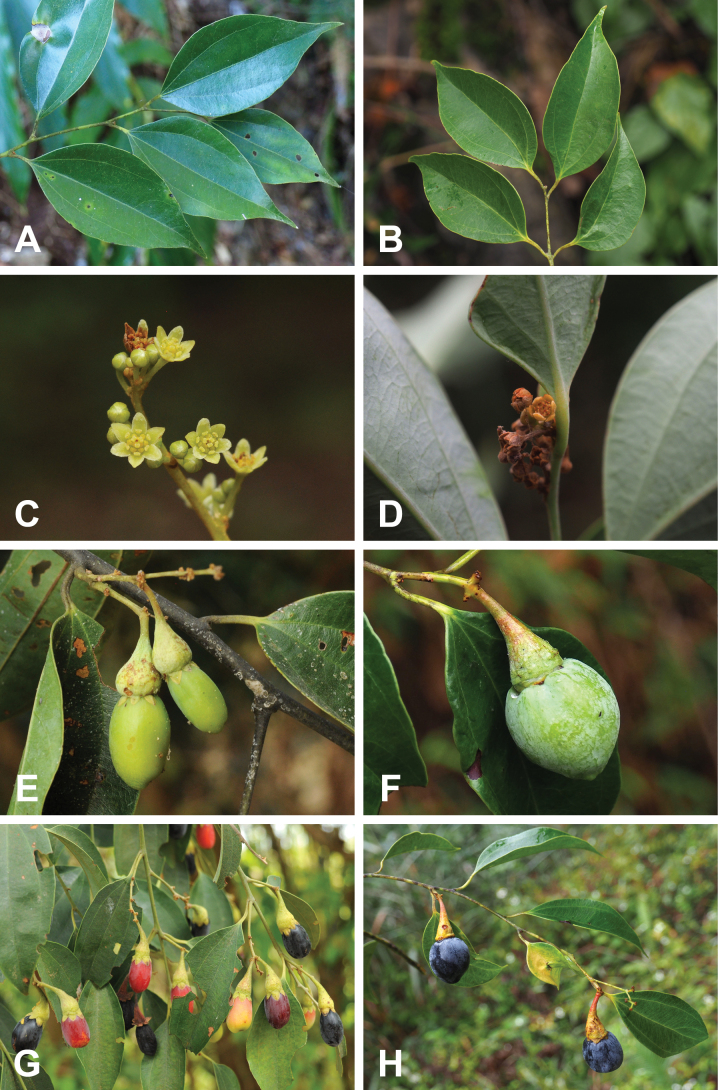
Side-by-side morphological comparison of *Neocinnamomum caudatum* (ACEG) and *Neocinnamomum macrocarpum* (BDFH). **A, B.** Branch and leaves; **C, D.** Inflorescence; **E, F.** Young fruits; **G, H.** Mature fruits.

#### Type.

**China. Guangxi Province** • Debao County, Dongling; 25 Nov 2014; fruit; *Shou-Jun Zhang & Wen-Bin Xu 140607* (holotype: HIB!).

#### Additional specimens examined.

**China. Guangxi Province** • Debao County, Dongling; 1 Aug 2016; flower; *Wen-Bin Xu & Bo-Shun Xia 160801* (paratype, HIB!), *Wen-Bin Xu & Bo-Shun Xia 160802* (paratype: HIB!); • Long’an County, Dujie, Sanle, Zhusha; 8 Dec 2024; fruit; *Bing Liu, Wei-Lan Lin, Zhi Yang 17840, 17842, 17843* (PE); • Long’an County, Dujie, Sanle, Zhusha; 14 May 2025; flower; *Bing Liu, Wei-Lan Lin, Zhi-Yong Tang 18478* (PE); • Debao County, Dongling, Duomo, Duoli; 9 Dec 2024; fruit; *Bing Liu, Wei-Lan Lin, Zhi Yang 17850, 17855* (PE); • Debao County, Dongling, Duole; 9 Dec 2024; fruit; *Bing Liu, Wei-Lan Lin, Zhi Yang 17856, 17857* (PE); • Pingguo City, Haicheng, Yongliang, Longhe; 21 Jan 2025; *Bing Liu, Wei-Lan Lin 18132* (PE); • Jingxi City, Ande, Xincun; 10 Nov 1956; *Yin-Kun Li P00834* (PE); • Jingxi City, Yuexu, Xiamin; 20 Aug 2021; *Xi-Tao Li, Huan Xiao ZYA01534* (PE); • Jingxi City, Huadong, Lixing; 1 Jun 2021; *Shi-Yue Nong, Qiu-Yan Lu ZYB00171* (PE); • Jingxi City, Huadong, Wuquan, Quantun; 10 Dec 2024; fruit; *Bing Liu, Wei-Lan Lin, Zhi Yang 17877, 17878, 17879* (PE); • Jingxi City, Huadong, Wuquan, Quantun; 15 May 2025; flower; *Bing Liu, Wei-Lan Lin, Zhi-Yong Tang 18517, 18519* (PE).

#### Notes.

Neocinnamomum caudatum var. macrocarpum was published as a variety of *N. caudatum*. The two taxa share several characters, such as puberulent branchlets, glabrous leaf blades with numerous transverse veins, and dark purple fruits when mature. However, morphological differences indicate that N. caudatum var. macrocarpum should be elevated to species rank, namely *N. macrocarpum*. The main differences between the two species lie in leaf blade venation, inflorescence structure, flower size, and fruit shape. *N. macrocarpum* differs from *N. caudatum* by its triplinerved leaves (vs. trinerved leaves), axillary cymules or cymules rarely arranged in terminal panicles (vs. numerous cymules arranged in both axillary and terminal panicles), small flowers (3–4 mm in diameter vs. 6–8 mm in diameter), and widely ovoid fruits (2–3 × 1.6–2.8 cm vs. narrowly ellipsoid fruits, 1.5–2 × 0.8–1 cm) (Table 1).

**Table 1.** A morphological comparison among *Neocinnamomum macrocarpum*, *N. caudatum*, *N. citratum*, and *N. lecomtei*.

Character	*N. macrocarpum*	*N. caudatum*	*N. citratum*	*N. lecomtei*
Branchlet	puberulent to glabrous	puberulent to glabrous	rusty or white pubescent but soon glabrous	densely pubescent initially but puberulent when mature
Leaf pubescence	glabrous on both surfaces	glabrous on both surfaces	sparsely white sericeous on abaxial surface when young and glabrous on both surfaces when mature	densely rusty pubescent on both surfaces when young and abaxially when mature
Leaf blade size (cm)	3–9 × 2.5–7	4–12 × 2–4.5	3.5–13 × 1.5–5.3	(5.5–) 8–12 × (2.5–) 4–7.5
Leaf vein	triplinerved	trinerved	trinerved	trinerved
Inflorescence	cymules axillary or rarely arranged in terminal panicles	panicle terminal and axillary	cymules axillary, rarely terminal	cymules axillary
Flower size (mm)	3–4	6–8	4–4.5	4–4.5
Tepals pubescence	rusty puberulent on two sides	rusty puberulent on two sides	densely rusty puberulent on two sides	densely rusty puberulent on two sides
Fruit shape	widely ovoid	narrowly ellipsoid	oblate to globose	ellipsoid
Fruit size (cm)	2–3 × 1.6–2.8	1.5–2 × 0.8–1	1.5–2 × 1.5–3	1.5–2.5 × 0.9–2

The native range of *N. macrocarpum* is considerably larger than its type locality. Based on our field investigations, this species is widely distributed in southwestern Guangxi (Fig. 5, red triangles). Moreover, *N. macrocarpum* grows on limestone mountains, whereas *N. caudatum* occurs on granite or other non-limestone mountains.

### ﻿ Neocinnamomum citratum

Taxon classificationPlantaeLauralesLauraceae

C.Y.Deng, Bing Liu & Wei L.Lin sp. nov.

E863C4D9-C339-5A29-BD73-AAEB71A75FB8

urn:lsid:ipni.org:names:77375096-1

Figs 2, 3, 4

#### Type.

**China. Guizhou Province** • Qianxinan Buyi and Miao Autonomous Prefecture, Xingyi City, Wanfenglin, Wanfu Village; alt. ca. 1178 m elev.; 17 Dec 2024; fruit; *Bing Liu, Wei-Lan Lin, Zhi Yang & Chao-Yi Deng 17921* (holotype: PE!; isotypes: PE!).

**Figure 2.**
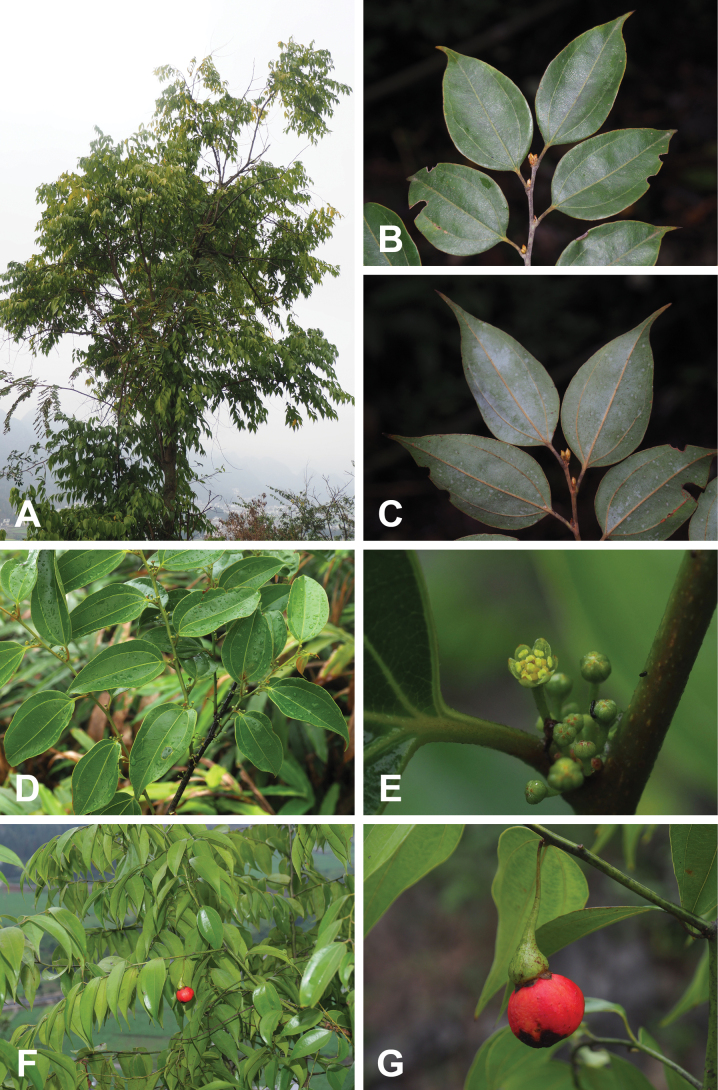
Morphology of *Neocinnamomum citratum* sp. nov. **A.** Habit; **B.** Adaxial view of leaf blade; **C.** Abaxial view of leaf blade; **D.** Flowering branch; **E.** Inflorescence; **F.** Fruiting branch; **G.** A fruit with persistent perianth lobes.

#### Diagnosis.

This new species is close to *N. lecomtei* but differs from sparsely white sericeous leaf blade on abaxial surface when young and glabrous when mature (vs. densely rusty pubescent on both surfaces when young and abaxially when mature) and oblate to globose fruits (vs. ellipsoid fruits), as well as its strong lemon fragrance of branches, leaves, and bark (vs. faint fragrance in the latter species).

**Figure 3.**
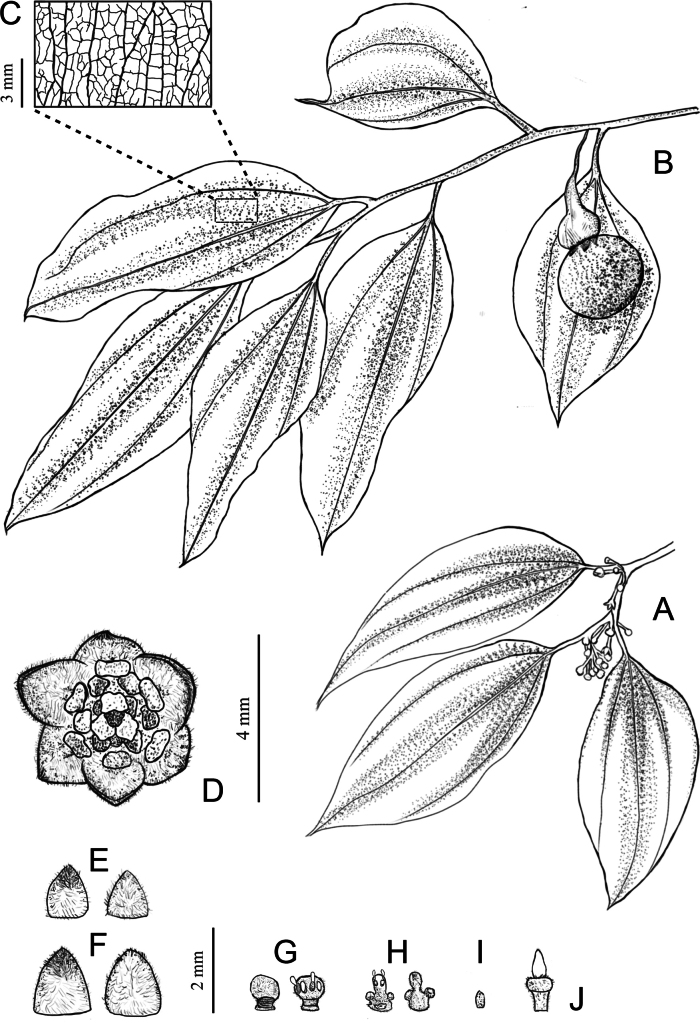
Illustrations of *Neocinnamomum citratum* sp. nov. showing morphological details. **A.** Flowering branch; **B.** Fruiting branch; **C.** Leaf blade venation; **D.** A flower showing the spreading perianth lobes and four whorls of stamens; **E.** Abaxial (left) and adaxial (right) views of outer perianth lobes; **F.** Abaxial (left) and adaxial (right) views of inner perianth lobes; **G.** Abaxial (left) and adaxial (right) sides of fertile stamens of the first and second whorls; **H.** Abaxial (right) and adaxial (left) sides of fertile stamens of the third whorl; **I.** The fourth-whorl staminode; **J.** Pistil. Drawn by Zi-Yi Zhang.

#### Description.

Trees, 4.5–20 m tall. Bark yellowish brown, old bark gray. Branchlets terete, rusty, or white pubescent initially but soon glabrous. Leaves alternate. Petiole 0.6–1.4 cm long, rusty, or white tomentose when young. Leaf blade papyraceous, narrowly ovate to ovate, 3.5–13 × 1.5–5.3 cm, apex acuminate, base acuminate to obtuse; sparsely white sericeous on abaxial surface when young and glabrous on both surfaces when mature, abaxially white farinose, trinerved, secondary lateral veins inconspicuous, veinlets densely reticulate. Inflorescences cymules, axillary, rarely terminal, with 2 to more than ten flowers, peduncle 0.2–4 mm long, pedicels 0.5–1.0 cm long, peduncle and pedicels rusty pubescent. Bracts subulate, 1.5 mm long, densely rusty puberulent. Flowers bisexual, yellowish green, ca. 4 mm in diam. Perianth lobes 6, subequal, triangular-ovate, ca. 1.2–1.5 mm long, densely rusty puberulent outside and inside. Fertile stamens 9, ca. 1 mm long, rusty puberulent; filaments as long as anthers, those of 3^rd^ whorl each with 2 glands, others glandless; those of 1^st^ and 2^nd^ whorls each with 2 introrse cells and 2 lateral cells, 4 oval cells arranged in an almost transverse series; those of 3^rd^ whorl each with 2 extrorse cells and 2 lateral cells. Staminodes ovoid, ca. 0.4 mm long, nearly estipitate. Ovary ellipsoid-ovoid, ca. 0.8 mm long. Style short, stigma discoid. Fruit oblate to globose, 1.5–2 × 1.5–3 cm, red when mature. Perianth cup in fruit crateriform, 0.6–1 cm wide on top; perianth lobes in fruit persistent.

**Figure 4.**
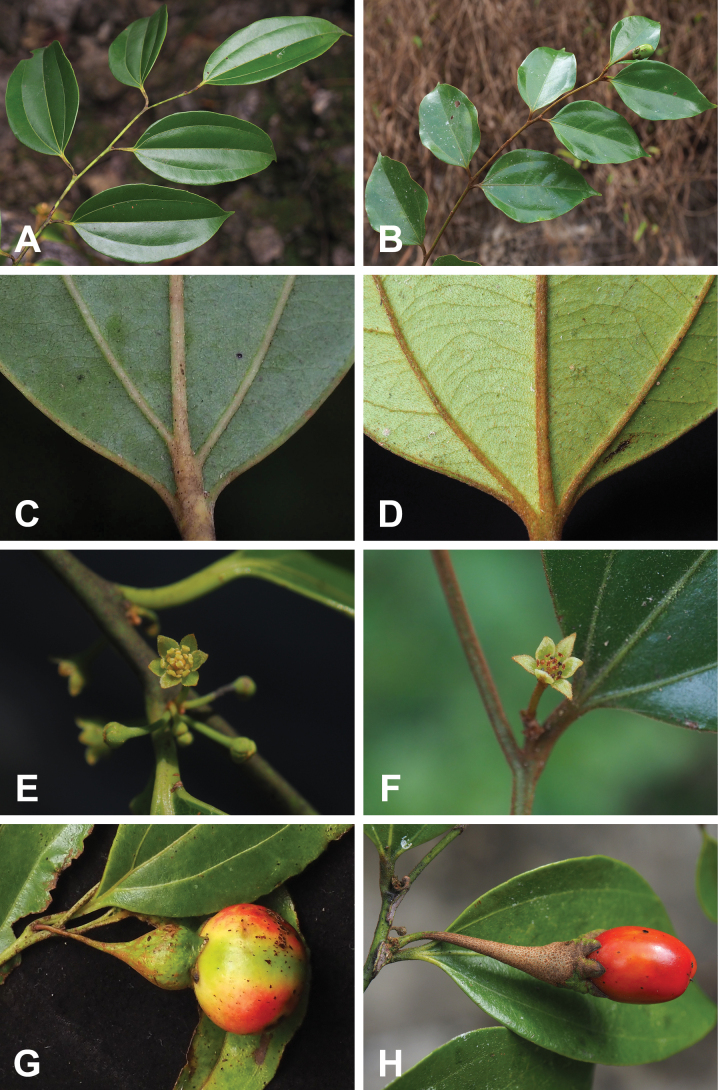
Side-by-side morphological comparison of *Neocinnamomum citratum* (ACEG) and *Neocinnamomum lecomtei* (BDFH). **A, B.** Branch and leaves; **C, D.** Abaxial surface of leaf blade base; **E, F.** Inflorescence; **G, H.** Mature fruits.

#### Distribution.

**Figure 5.**
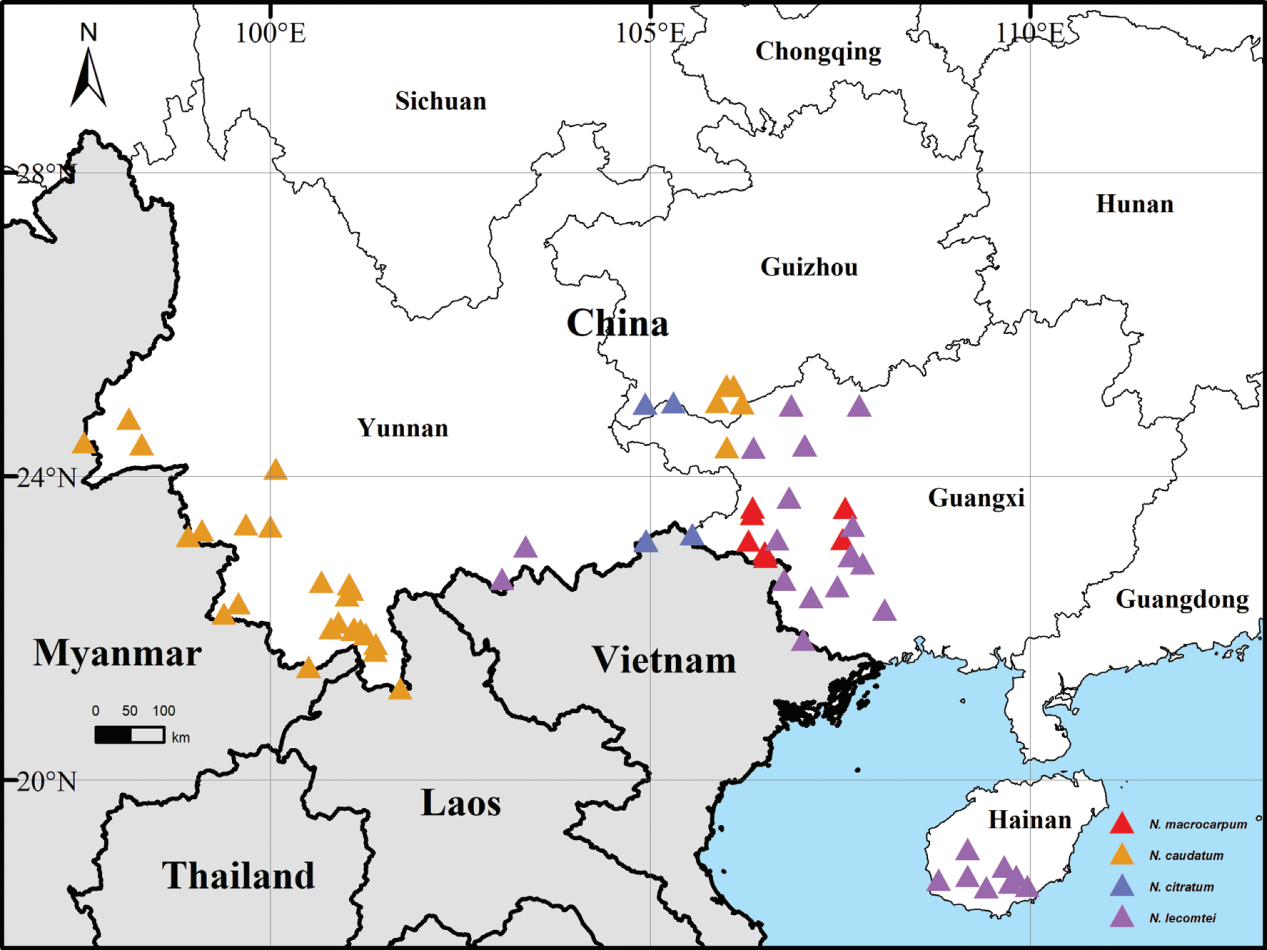
Map showing the distribution of *Neocinnamomum caudatum* (yellow), *N. macrocarpum* (red), *N. citratum* (blue), and *N. lecomtei* (purple).

Southwestern China (Guangxi, Guizhou, Yunnan) (Fig. 5, blue triangles).

#### Habitat.

This species occurs in evergreen forests of limestone mountains with an altitudinal range of 1000 to 1300 m. It blooms in May and June, and the fruits are mature during September and December.

#### Etymology.

The specific epithet “*citratum*” refers to the strong lemon fragrance of the branches, leaves, and bark of this species.

#### Additional specimens examined (paratypes).

**CHINA. Guangxi Province** • Napo County, Baidu, Nonghua, Nongbu; 16 May 2025; flower; *Bing Liu, Wei-Lan Lin, Zhi-Yong Tang 18531* (PE!, KUN!, CSH!), *18535* (PE!). **Guizhou Province** • Anlong County, Dewo, Tingxi; 18 Sep 1991; fruit; *Chao-Yi Deng 50462* (KUN!); • Anlong County, Dewo, Dewo Village; 28 April 2025; flower; *Chao-Yi Deng 20250428001* (XIN!); • Xingyi City, Wanfenglin, Wanfu Village; 11 May 2025; flower; *Chao-Yi Deng 20250511001* (XIN!, PE!, KUN!); • Anlong County, Panlong, Yangjiawan; 13 May 2025; cultivated, flower; *Bing Liu, Wei-Lan Lin, Zhi-Yong Tang 18456* (PE!). **Yunnan Province** • Malipo County, Babu, Nadeng, Danong; 14 Oct 2010; fruit; *Bing Liu 1175* (PE!); • Malipo County, Babu, Nadeng, Danong; 15 Dec 2024; fruit; *Bing Liu, Wei-Lan Lin, Zhi Yang, Chao-Yi Deng 17901* (PE!, KUN!, CSH!); • Malipo County, Babu, Nadeng, Danong; 18 May 2025; flower; *Bing Liu, Wei-Lan Lin, Zhi-Yong Tang 18563* (PE!, KUN!, CSH!), *18565* (PE!, CSH!), *18567* (PE!).

#### Uses.

The branches, leaves, and bark of *Neocinnamomum citratum* have a strong lemon fragrance. Therefore, its leaves and branches are collected by the local people in Malipo County and used as spices for cooking.

#### Conservation.

There are five populations with more than 100 individuals. Based on IUCN Red List Categories and Criteria (IUCN 2024), the new species is categorized as “Not Threatened” (NT Blab (v) + D).

#### Notes.

The new species has a strong lemon fragrance in its branches, leaves, and bark, whereas other species of *Neocinnamomum* have only a faint fragrance. The rusty pubescence on the branchlets and the sparsely white sericeous pubescence on the abaxial surface of the leaf blade when young, becoming glabrous when mature, distinguish it from *N. caudatum*, *N. fargesii*, *N. macrocarpum*, and *N. mekongense*, which are nearly glabrous, as well as from *N. delavayi* and *N. lecomtei*, which are densely pubescent on both leaf surfaces when young and abaxially when mature. The oblate to globose fruits also distinguish this species from the ellipsoid to narrowly ellipsoid fruits of *N. caudatum* and *N. lecomtei* and from the ovoid to ellipsoid-ovoid fruits of *N. fargesii*, *N. delavayi*, *N. macrocarpum*, and *N. mekongense* (Table 1).

## Supplementary Material

XML Treatment for Neocinnamomum macrocarpum

XML Treatment for Neocinnamomum citratum

## ﻿Additional information

### Use of AI

No use of AI was reported.

### Author ORCIDs

Wei-Lan Lin https://orcid.org/0009-0008-6997-9540

Zi-Yi Zhang https://orcid.org/0009-0004-7717-7522

Chao-Yi Deng https://orcid.org/0000-0001-5407-5292

Wen-Bin Xu https://orcid.org/0009-0002-2645-0053

Bing Liu https://orcid.org/0000-0002-6086-253X

### Data availability

All of the data that support the findings of this study are available in the main text.
